# Perceptions and Prevalence of Cannabis Use in Women With Inflammatory Bowel Disease of Reproductive Age: A Cross-Sectional Study

**DOI:** 10.1093/jcag/gwad049

**Published:** 2023-11-25

**Authors:** Nariman Hossein-Javaheri, Katie O’Connor, Hillary Steinhart, Amol Deshpande, Cynthia Maxwell, Vivian Huang, Parul Tandon

**Affiliations:** Department of Internal Medicine, University at Buffalo-State University of New York, Buffalo, New York, USA; Division of Gastroenterology and Hepatology, University Health Network/Mount Sinai Hospital, University of Toronto, Toronto, Ontario, M5G 1X5Canada; Division of Gastroenterology and Hepatology, University Health Network/Mount Sinai Hospital, University of Toronto, Toronto, Ontario, M5G 1X5Canada; Department of Family and Community Medicine, Toronto Rehabilitation Institute, Quality and Innovation, University of Toronto, Ontario, Canada; Department of Obstetrics and Gynecology, Mount Sinai Hospital, University of Toronto, Ontario, Canada; Division of Gastroenterology and Hepatology, University Health Network/Mount Sinai Hospital, University of Toronto, Toronto, Ontario, M5G 1X5Canada; Division of Gastroenterology and Hepatology, University Health Network/Mount Sinai Hospital, University of Toronto, Toronto, Ontario, M5G 1X5Canada

**Keywords:** IBD, cannabis, pregnancy, ulcerative colitis, Crohn’s disease

## Abstract

**Background:**

Many patients with inflammatory bowel disease (IBD) may use cannabis for relief of symptoms. During pregnancy, however, cannabis exposure may be associated with adverse pregnancy outcomes. We aimed to determine the prevalence and perceptions of cannabis use in women with IBD.

**Methods:**

Through recruitment at Mount Sinai Hospital and online platforms such as Twitter, women with IBD (age 18–45) were asked to complete anonymous surveys on demographics, cannabis use, perception of use during pregnancy, and discussing its use with healthcare providers (HCP). Categorical variables were reported as frequencies and compared across groups with the chi-square test.

**Results:**

One-hundred and two pregnant patients with IBD were included in this study, 19 (18.6%) reported using cannabis. Current users were more likely to report constant pain in the last 12 months and discuss its use with their HCP. Fifty-three (52.0%) women were unsure of the specific risks associated with cannabis use during pregnancy, and only 15 (14.7%) had ever discussed its use with their HCP. Those who had discussed cannabis use with their HCP were more likely to have prior IBD-related surgery, perceive its use unsafe during pregnancy, and be more likely to be using cannabis.

**Conclusion:**

Many women with IBD report uncertainty of the risks of cannabis use during pregnancy and the majority have never discussed cannabis use with their providers. With the increasing legalization of cannabis in many jurisdictions, it is imperative patients and healthcare providers discuss the risks and benefits of its use, particularly during vulnerable times such as pregnancy.

## Introduction

Inflammatory bowel disease (IBD) is characterized by chronic inflammation of the gastrointestinal (GI) tract. The two major types of IBD are Crohn’s disease (CD) and ulcerative colitis (UC). IBD may occur in genetically susceptible individuals and can occur at a prevalence of up to 0.7 percent in countries such as Canada.^1^ Due to the chronic nature of the disease, many patients experience periods of debilitating pain which in turn negatively impacts quality of life.^2^ Consequently, up to 70 percent of patients with IBD may use medications such as non-steroidal anti-inflammatories (NSAID) and acetaminophen to manage these symptoms.^2,3^

Some use complementary and alternative medicines (CAM) such as cannabis to manage symptoms related to IBD.^3,4^ Cannabis contains a variety of ingredients including cannabidiol (CBD) and tetrahydrocannabinol (THC)^5^ and though its use may not induce clinical remission in IBD,^6^ there can be significant improvements in abdominal pain, diarrhea, and quality of life.^3,4^ It is estimated that up to 50 percent of IBD patients have used cannabis at some point during the course of their disease.^7,8^ This appears to be higher than the general population where the estimated prevalence of use is approximately 25 percent.^9^ There remain however certain risks associated with cannabis use including decreased adherence to medications, fewer follow-ups with care providers, and increased rates of hospitalization.^8^

The greatest incidence of cannabis use occurs in ages 20–24 in the general population,^9,10,^ and on average, this is about 4 years earlier in patients living with IBD.^11^ This then has implications for women of reproductive age who may be using cannabis during preconception and pregnancy. *In utero* cannabis, exposure may cause adverse pregnancy outcomes such as gastroschisis, neonatal growth restrictions, preterm birth, and potential neurodevelopmental consequences.^12–14^ Women with IBD, particularly those with active disease, are also at an inherently increased risk of worsened pregnancy outcomes such as miscarriage, infants born small for gestational age, preterm prelabour rupture of membranes, and emergent caesarean deliveries.^15–19^ It remains unknown how cannabis use further impacts the risk of these adverse outcomes in IBD.

The estimated prevalence of cannabis use during pregnancy in IBD remains unknown.^20,21^ Understanding this is likely the first step to determine the potential negative synergistic impacts of cannabis use on adverse pregnancy outcomes in IBD patients. Hence, this study aims to characterize both the perception and prevalence of cannabis use during preconception and pregnancy in women with IBD.

## Methods

This cross-sectional study was approved by the institutional review and Human Research Ethics Board of Mount Sinai Hospital in Toronto, Canada. All study participants provided informed consent before completing the questionnaires and responses were anonymous.

### Study design and outcomes

Women aged 18–45 with IBD (both UC and CD) were recruited through a dedicated IBD and pregnancy clinic at Mount Sinai Hospital with a survey response rate of approximately 50%, and social media platforms including Twitter, Facebook, and the Crohn’s and Colitis Canada website. Participants were provided multiple anonymous surveys and were asked to provide information regarding baseline demographics, IBD characteristics, other comorbidities, and pain symptoms. In addition, participants were provided a questionnaire on the current use of cannabis, and perceptions of its use, during pregnancy.

### Survey content

The demographics survey included current age, annual gross household income, marital status, employment status, and life stages such as preconception, currently pregnant, or postpartum (delivered an infant in the 12 months prior to survey completion). The IBD survey included the type of IBD (UC, CD, or indeterminate colitis), the year of diagnosis, and whether the participants had prior surgery related to their IBD. Next, we asked the participants to report the presence of comorbidities such as mental health illnesses (i.e., anxiety and depression), irritable bowel syndrome (IBS), and other conditions diagnosed prior to survey completion. All findings were self-reported by the participants.

Participants were asked to complete a pain survey and to report on pain symptoms over the last year prior to survey completion (no pain at all, pain with IBD flares and fine in between, and constant pain). They were also asked to report the average level of pain over the last two weeks prior to survey completion (from a scale of 0–10 with 0 representing no pain and 10 representing severe pain).

Finally, we also asked the participants to report on perceptions and current use of cannabis. Specific methods of cannabis use, such as inhalation versus edible intake, were not specifically addressed. Participants were asked if they had ever discussed cannabis use with their healthcare provider (HCP) (yes vs. no), whether they were current users of cannabis at the time of survey completion (yes vs. no), and whether they considered active use of cannabis during pregnancy to be safe or unsafe. If they felt it was unsafe, they were asked to comment on specific reasons why they felt this way (which included restricted oxygenation to the fetus, respiratory problems such as asthma, immune disorders such as eczema, or impaired brain development). Finally, they were asked to comment on any concurrent uses of nicotine, alcohol use, or recreational drugs at the time of survey completion (Supplemental Document 1).

### Statistical analysis

Descriptive statistics were reported as medians with interquartile ranges (IQR) for continuous variables or frequencies with percentages for categorical variables. Continuous variables were compared across groups using the non-parametric Mann–Whitney-*U* test whereas categorical variables were compared using the chi-square test. All tests were reported as two-sided, with *P* values < 0.05 considered statistically significant. Analysis was performed using the SPSS software version 26 (Chicago, IL). Given the primary purpose of the study was descriptive and hypothesis-generating, no correction for multiple comparisons was performed.

## Results

Overall, 102 women with IBD were included in this study: 42 with UC (41.2%), 57 with CD (55.9%), and three with indeterminate colitis (2.9 percent). Forty (39.2 percent) women were not pregnant, 57 (55.9 percent) were pregnant, and five (4.9 percent) were post-partum. Most women were married (*n* = 81, 79.4 percent), employed (*n* = 90, 88.2 percent), with a household income greater than $100,000 (*n*=54, 52.9 percent), and with Canadian nationality (*n* =78, 76.5 percent). With respect to prior comorbidities, 22 patients (21.6 percent) reported a history of depression, 28 (27.5 percent) a history of anxiety, and 29 (28.4 percent) a history of IBS. Additional baseline characteristics are described in Table 1.

**Table 1.** Baseline demographics of included studies

Characteristic	Total (*n* = 102)
Age	
21–30	26 (25.5%)
31–40	70 (68.6%)
41–50	9 (5.9%)
IBD type	
UC	42 (41.2%)
CD	57 (55.9%)
IBD-U	3 (2.9%)
Prior IBD surgery	
No	76 (74.5%)
Yes	26 (25.5%)
Comorbidities	
Depression	22 (21.6%)
Depression symptoms in the last 2 weeks	22 (21.6%)
Anxiety	28 (27.5%)
Anxiety symptoms in the last 2 weeks	36 (35.3%)
IBS	29 (28.4%)
Marital status	
Single	9 (8.8%)
Married	81 (79.4%)
Common-law	9 (8.8%)
Separated	2 (2.0%)
Missing	1 (1.0%)
Nationality	
Canadian	78 (76.5%)
American	5 (4.9%)
Afghan	1 (1.0%)
Brit	4 (3.9%)
Iranian	1 (1.0%)
Indian	1 (1.0%)
Non-cannabis recreational drug use	
None	70 (68.6%)
Alcohol	22 (21.6%)
Cigarettes	2 (2.0%)
Other recreational drugs	5 (4.9%)
Smoking and alcohol	3 (2.9%)
Work status	
Employed	90 (88.2%)
Unemployed	12 (11.8%)
Income	
<$20,000	2 (2.0%)
$20,000–49,999	9 (8.8%)
$50,000–79,999	12 (11.8%)
$80,000–99,999	22 (21.6%)
$100,000 <	54 (52.9%)
Prefer not to say	3 (2.9%)

Nine (8.8 percent) women reported experiencing no pain in the 12 months prior to survey completion, 61 (59.8 percent) reported pain only with IBD flares, and 32 (31.4 percent) reported constant pain. The median overall pain score in the two weeks prior to survey completion was 2.0 (range 1.0–10.0). Twenty-two (21.6 percent) and 36 (35.3 percent) women reported experiencing depression and anxiety symptoms in the 2 weeks prior to survey completion respectively.

### Prevalence of cannabis use

**Figure 1.**
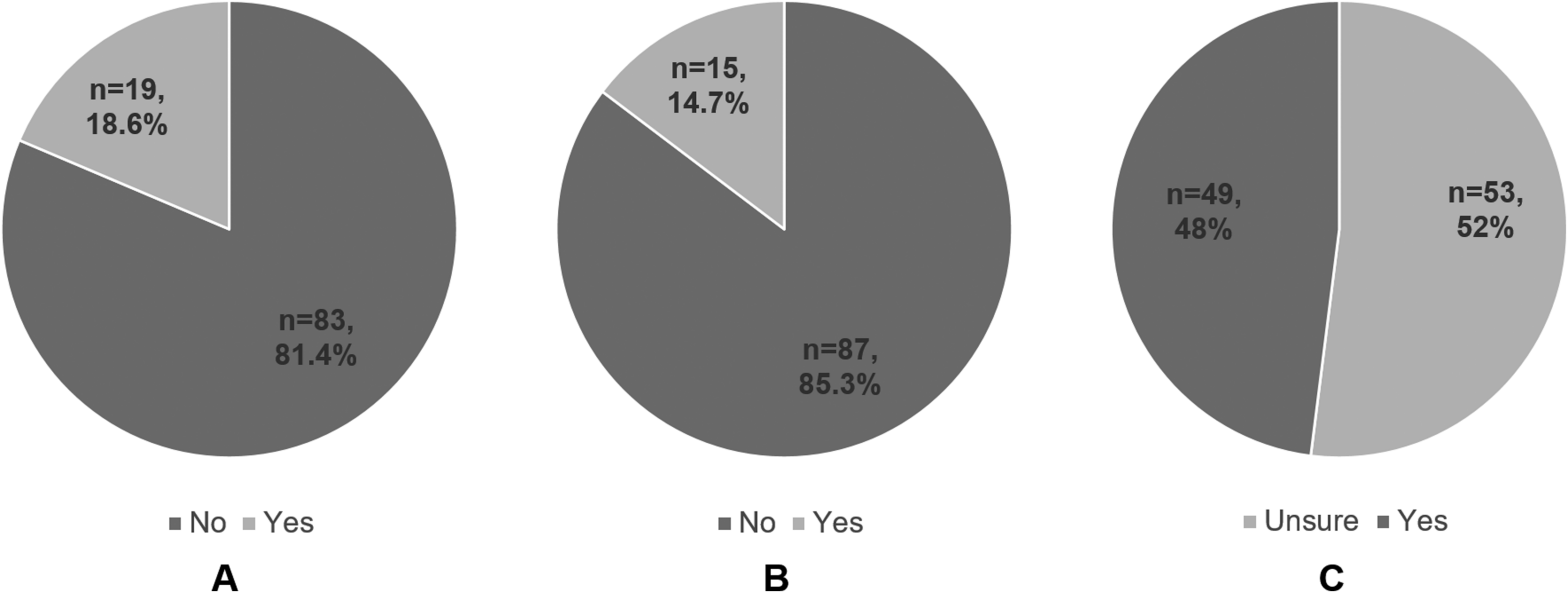
(A) Percentage of cannabis users at the time of the survey. (B) Percentage of patients who were aware of the risks of cannabis during pregnancy. (C) Percentage of patients who had discussed cannabis use with their health-care providers (HCP).

Nineteen (18.6 percent) participants reported using cannabis at the time of the survey, 13 were preconception and 6 were pregnant (fig. 1A). Those reporting current use were more likely to report constant pain in the prior 12 months (21.1 percent vs. 6.0 percent, *P* = 0.01) and were more likely to perceive that its use is safe during pregnancy (15.8 percent vs. 1.3 percent, *P* = 0.003). There was no difference in IBD subtype, disease duration, prior IBD surgery, mental health disorders, income and employment status, and marital status between users and non-users of cannabis (Table 2).

**Table 2.** Cannabis users vs. non-users.

	Non-cannabis users *n* = 83 (81.4%)	Cannabis users *n* = 19 (18.6%)	*P*
Pregnancy stage			0.01
Preconception	27 (32.5%)	13 (68.4%)	
Pregnant	51 (61.4%)	6 (31.6%)	
Postpartum	5 (6.0%)	0 (0%)	
IBD type			0.13
UC	38 (45.8%)	4 (21.1%)	
CD	43 (51.8%)	14 (73.7%)	
Indeterminate colitis	2 (2.4%)	1 (5.3%)	
IBD disease duration	10.50 (IQR 10)	14 (IQR 9.50)	0.26
Prior IBD surgery	20 (24.1%)	6 (31.6%)	0.50
Pain experience			0.01
No pain	5 (6.0%)	4 (21.1%)	
Pain with IBD flares	20 (24.1%)	6 (31.6%)	
Constant pain	5 (6.0%)	4 (21.1%)	
Two-week pain score (median)	2.00 (IQR 3.00)	3.00 (IQR 6.00)	
Diagnosis of depression	18 (21.7%)	4 (21.1%)	0.95
Depression symptoms in the last two weeks	17 (26.6%)	5 (29.4%)	0.81
Diagnosis of anxiety	21 (25.3%)	7 (36.8%)	0.31
Anxiety symptoms in the last 2 weeks	25 (39.1%)	11 (64.7%)	0.06
Diagnosis of IBS	21 (25.3%)	8 (42.1%)	0.14
Is the patient aware of the potential side effects of cannabis during pregnancy?			0.34
Yes	34 (43.0%)	10 (55.6%)	
No	45 (57.0%)	8 (44.4%)	
Is cannabis use safe during pregnancy?			0.005
Yes	1 (1.3%)	3 (15.8%)	
No	60 (75.0%)	15 (78.9%)	
Not sure	19 (23.8%)	1 (5.3%)	
Patients who discussed cannabis use with their healthcare provider	8 (9.6%)	7 (36.8%)	0.003
Age			0.48
21–30	21 (25.3%)	5 (26.3%)	
31–40	56 (67.5%)	14 (73.7%)	
41–50	6 (7.2%)	0 (0)	
Income			0.45
<$20,000	1 (1.2%)	1 (5.3%)	
$20,000–49,999	8 (9.6%)	1 (5.3%)	
$50,000–79,999	10 (12.0%)	2 (10.5%)	
$80,000–99,999	20 (24.1%)	2 (10.5%)	
$100,000 or more	41 (49.4%)	13 (68.4%)	
Marital status			0.15
Single	5 (6.04%)	4 (21.1%)	
Married	66 (79.5%)	15 (78.9%)	
Common law	9 (10.8%)	0 (0%)	
Separated	2 (2.4%)	0 90%)	
Work status			0.16
Employed	75 (90.4%)	15 (78.9%)	
Unemployed	8 (9.6%)	4 (21.1%)	

### Perceptions of cannabis safety during pregnancy

Regarding perceptions of cannabis use during pregnancy, 75 (73.6 percent) considered its use unsafe, 20 (19.6 percent) reported uncertainty about its safety, and 4 (3.9 percent) felt it was safe to use throughout pregnancy. Fifty-three (52.0 percent) women were unsure of the specific risks associated with cannabis use during pregnancy (fig. 1B). Those that reported being aware of the specific risks identified risks of neurocognitive delay (*n* = 38, 37.3 percent), risk of foetal respiratory illness (*n* = 22, 21.6 percent), risk of neonatal eczema (*n* = 2, 2.0 percent), and risk of foetal oxygen restriction (*n* = 19, 18.6 percent).

### Cannabis discussions with HCP

Fifteen (14.7 percent) participants recalled ever having a discussion of cannabis use with their HCP (fig. 1C). Those who had discussed cannabis use with their HCP were more likely to have a prior IBD surgery (53.3 percent vs. 20.7 percent, *P* = 0.007), were more likely to perceive cannabis use as unsafe during pregnancy (100 percent vs. 71.4 percent, *P* = 0.005), and were more likely to be current users of cannabis (46.7 percent vs. 13.7 percent, *P* = 0.003). Those who did not discuss cannabis use with their HCP were less aware of its safety risks during pregnancy; 60 (71.4 percent) considered it unsafe, 20 (23.8 percent) were uncertain of its safety and 4 (4.8 percent) said it was safe. Only 35 (42.7 percent) of these participants were aware of potential side effects. The life stage, IBD type, disease duration, other comorbidities, and the presence of pain were not significantly different amongst the two groups (Table 3).

**Table 3.** Participants who discussed cannabis use with their HCP.

	Never discussed cannabis use *n* = 87 (85.3%)	Discussed cannabis use *n* = 15 (14.7%)	*P*
Pregnancy stage			0.63
Preconception	34 (39.1%)	6 (40.0%)	
Pregnancy	48 (55.2%)	9 (60.0%)	
Postpartum	5 (5.7%)	0 (0%)	
IBD type			0.16
UC	39 (44.8%)	3 (20.0%)	
CD	46 (52.9%)	11 (73.3%)	
Indeterminate colitis	2 (2.3%)	1 (6.7%)	
IBD disease duration	11.00 (10.0)	12.00 (12.00)	0.69
Prior IBD surgery	18 (20.7%)	9 (53.3%)	0.007
Pain experience			0.94
No pain	8 (9.2%)	1 (6.7%)	
Pain with IBD flares	52 (59.8%)	9 (60.0%)	
Constant pain	27 (31.0%)	5 (33.3%)	
Median pain score in the last 2 weeks	2.00 (3.00)	2.00 (5.00)	
Diagnosis of depression	18 (20.7%)	4 (26.7%)	0.60
Depression symptoms in last 2 weeks	19 (26.8%)	3 (30.0%)	0.31
Diagnosis of anxiety	23 (26.4%)	5 (33.3%)	0.58
Anxiety symptoms in the last 2 weeks	30 (42.3%)	6 (60.0%)	0.29
Diagnosis of IBS	25 (28.7%)	4 (26.7%)	0.87
Is patient aware of the potential side effects of cannabis during pregnancy?			0.22
Yes	35 (42.7%)	9 (60.0%)	
No	47 (57.3%)	6 (40.0%)	
Is cannabis use safe during pregnancy?			0.05
Yes	4 (4.8%)	0 (0%)	
No	60 (71.4%)	15 (100%)	
Not sure	20 (23.8%)	0 (0%)	
Age			0.46
21–30	23 (26.4%)	3 (20.0%)	
31–40	58 (66.7%)	12 (80.0%)	
41–50	6 (6.9%)	0 (0%)	
Income			0.52
<$20,000	2 (2.3%)	0 (0%)	
$20,000–49,999	9 (10.3%)	0 (0%)	
$50,000–79,999	11 (12.6%)	0 (0%)	
$80,000–99,999	17 (19.5%)	5 (33.3%)	
$100,000 or more	46 (52.9%)	8 (53.3%)	
Marital status			0.91
Single	7 (8.0%)	2 (13.3%)	
Married	69 (79.3%)	12 (80.0%)	
Common law	8 (9.2%)	1 (6.7%)	
Separated	2 (2.3%)	0 (0%)	
Work status			0.84
Employed	77 (88.5%)	13 (86.7%)	
Unemployed	10 (11.5%)	2 (13.3%)	
Cannabis use			0.003
Current user	12 (13.7%)	7 (46.7%)	
Non user	75 (86.3%)	8 (53.3%)	

## Discussion

In this cross-sectional study, we explored the prevalence and perception of cannabis use among women of reproductive age with IBD. We demonstrate that about one in five women with IBD report consuming cannabis and the majority report experiencing pain either constantly or with IBD flares in the last 12 months. Furthermore, though many considered cannabis use during pregnancy unsafe, the majority were unaware of its specific risks. Finally, very few women had ever discussed the use of cannabis during pregnancy with their HCP.

The estimated prevalence of cannabis use has increased from 15 percent in 2017 to 25 percent in 2021, likely in part due to legalization across numerous jurisdictions.^9^ Approximately 10 percent of women have reported use of cannabis, 4.2 percent during pregnancy, and 5.5 percent postpartum.^21,22^ Within pregnancy, variations have been observed with rates as high as 15.9 percent of women using cannabis during the reproductive years after legalization.^21^ This in turn has implications in IBD where previous studies have reported a prevalence of cannabis use as high as 50 percent.^23^ Our study demonstrates that about 19 percent of all women and 10 percent of pregnant women with IBD report cannabis use. Given the recent legalization of cannabis in Canada, the prevalence of use is likely increasing, and in regions with restricted access, use might be lower or underreported.

Reasons for cannabis use in the general population during pregnancy have been previously explored and include stress relief, improvement in first-trimester nausea or vomiting, and minimizing symptoms of chronic diseases such as pain.^18,21^ With IBD, even though cannabis use may not impact the inflammatory burden,^7,8,23^ its use has been reported to improve diarrhoea, abdominal pain, and appetite. However, those who consume cannabis may also be more likely to report frustration with their disease process, the inability of conventional medications to control their symptom burden, and a worse quality of life.^3^ Many then turn to alternative therapies, such as cannabis for symptom control. This is consistent with our study as those reporting cannabis use were more likely to report constant pain and prior IBD surgery which may be a surrogate for more complex disease.

Although many of the survey participants considered cannabis use unsafe during pregnancy, more than half were uncertain of its specific risks. Cannabinoids can cross the placenta and induce a variety of physiological effects on the foetus.^15^ Exposure may impact birth weight, length, and head circumference, increase rates of neonatal intensive care unit admissions, and infer a 2.9-fold risk of neonatal oxygen use.^24,25^ In addition, cannabis may induce epigenetic modifications which alter dopaminergic and cannabinoid pathways of the developing brain resulting in neuropsychiatric disorders and memory impairments in later life.^21,23,26^ From an obstetrical perspective, cannabis has been associated with higher rates of caesarean and preterm births.^24,26^ These risks also occur due to active disease in patients with IBD.^15–18^ It remains to be determined whether cannabis use particularly in IBD synergistically increases the risk of these adverse pregnancy outcomes.

In our study, a remarkably low percentage of women had discussed cannabis use with their healthcare providers. This is consistent with previous studies which have demonstrated that patients generally prefer avoiding such conversations.^26^ Although pregnant women may desire a better understanding of the effects of cannabis on the foetus, many might be hesitant to discuss its use due to fear of stigmatization, social desirability, and legalization status.^21,27,28^ Although studies on cannabis exposure during pregnancy rely on subjective reports and can be confounded by recall bias,^29^ many patients report receiving mixed or conflicting information about the safety of cannabis use from their providers and therefore are unable to come to any definitive conclusions.^27,28,30^

We conclude there is a critical need for educational resources and standard delivery of evidence-based information on the use of cannabis during pregnancy. A non-biased and respectful attitude focused on education is required to create an environment that facilitates discussion between patients and physicians.^26^ Although standardized educational resources are scarce, the Society of Obstetricians and Gynecologists of Canada has developed an interactive resource including videos and posters on cannabis use during pregnancy and lactation.^31^ Such resources that allow direct patient involvement with their care may serve as a valuable tool to spread awareness among patients and healthcare providers.

To our knowledge, this is the first study to specifically address cannabis use in pregnancy among women with IBD of reproductive age. Although we have not described specific reasons for cannabis use in this cohort of patients, our results suggest that current users experience greater pain and number of IBD surgeries compared to non-users. Our study does have inherent limitations. Our sample size remains small, and recruitment primarily occurred from a large tertiary care centre in Canada as well as online platforms which may lead to decreased external validity. However, an estimated 30–50 percent of patients report chronic pain even during disease remission.^32^ In our study, approximately 30 percent of participants reported constant pain. Although the reported percentage may not fully represent the general population, it could be utilized as an estimation of chronic pain and cannabis use in IBD patients.

To encourage participation, all surveys were collected anonymously limiting our ability to fully review the participant’s past IBD history, comorbidities, and delivery outcomes. Since all findings were self-reported, the possibility of recall bias cannot be excluded. In our survey, we did not specifically inquire how cannabis was consumed. Modes of ingestion may directly affect maternal health.^33^ Harm reduction options include minimizing exposure through smoking.^33^ Yet, overall exposure via edibles and vaporizers can affect the foetus since its metabolites readily cross the placenta.^18^ Future studies should explore the mode of Cannabis use on overall outcomes.

Furthermore, we did not differentiate between medical and recreational cannabis use. Considering its legalization in many jurisdictions, it is likely that most patients were utilizing recreational cannabis. While there is variability in its CBD concentrations, the THC levels of medical and recreational cannabis are similar.^34^ Approximately 5–15 percent of medical and 0–40 percent of recreational cannabis contains CBD.^34^ A greater CBD concentration can negatively impact the developing foetus.^35^ Therefore, use of medical versus recreational cannabis use should be investigated in the future.

In addition, we did not collect objective data to assess the disease severity of patients. Utilizing patient-reported outcomes (PROs) is beneficial in assessing perceived disease severity in IBD. Most PROs have included abdominal pain and stool frequency as markers of disease severity.^36^ Other objective approaches include use of endoscopic and laboratory markers such as faecal calprotectin.^37^ Yet, abdominal pain remains the primary reason for cannabis use.^3,4^ Although we did not obtain the frequency of bowel movements, we inquired about pain experience and generated a two-week pain score for a better understanding of patient experience. Since abdominal pain may persist independent of disease activity, some may continue cannabis use even with disease remission.^32^ Future studies should explore a potential association between disease severity and cannabis use in patients with IBD.

Due to the lack of a control group, we were also unable to directly compare the prevalence of cannabis use in IBD vs non-IBD patients. However, studies have shown that a greater percentage of IBD patients consume cannabis compared to the general population at similar rates demonstrated in our study.^7,8^ Furthermore, as inherent in observational cross-sectional qualitative research, there remains a risk of selection bias within our study. It is possible that patients participating in the study are more likely to discuss cannabis and its use with their HCP, but it is reasonable to assume that even fewer patients have such discussions with their providers than our study suggests. Finally, it is known that depression and anxiety are common mental health disorders among IBD patients.^38^ Although we asked participants about symptoms of depression and anxiety prior to the survey, we were not able to specifically consider mental health as a contributor to cannabis use due to a small sample size and lack of statistical power.

Overall, our study highlights the real-world experiences and perceptions of cannabis use in women living with IBD. Many patients use cannabis for symptomatic control of their IBD but report being unsure of the specific adverse effects of its use during high-risk periods such as pregnancy. Due to the potential risks of cannabis on the foetus and mothers, healthcare providers should implement a systemic screening approach and routinely discuss its use with their patients in the ambulatory setting.

## Supplementary Material

gwad049_suppl_Supplementary_Materials

## Data Availability

The raw data underlying this article cannot be shared publicly as participants did not provide consent for raw data sharing. The raw datasets generated during and/or analyzed during the current study are proprietary to Mount Sinai Hospital. However, the analyzed data protective of patient identity are available in the article.
