# Supplementary material for: Perceptions and Prevalence of Cannabis Use in Women With Inflammatory Bowel Disease of Reproductive Age: A Cross-Sectional Study
Source: J Can Assoc Gastroenterol. 2023 Nov 25;7(2):204–11. doi: 10.1093/jcag/gwad049 (PMC10999762; doi:10.1093/jcag/gwad049)
Supplement: gwad049_suppl_Supplementary_Materials [file gwad049_suppl_supplementary_materials.zip › Survey - upload.pdf]

Pain, Mood and IBD and Pregnancy Questionnaire – Pilot Study

Study ID#: ____________________________ Date: _________

Thank you for offering to complete this 15-20 minute survey today. All responses will be kept anonymous.

We are looking to understand how Inflammatory Bowel Disease (IBD) affects pregnancy, the things patients may use to manage their symptoms during this time-period, and how we might be better able to help patients manage their symptoms during pregnancy.

Throughout this survey, the term Cannabis will refer to Cannabis based products and/or Cannabinoids.

Thanks again for your time!

[THIS SECTION INTENTIONALLY LEFT BLANK]

SECTION A - Demographics

1. Which category best describes your current age?
a. 18-20
b. 21-30
c. 31-40
d. 41-50

2. What is your current body weight (indicate kg or lbs) ___________________

3. What is your current body height (cm or inches) ______________________

4. What is your current marital status?
a. Single (never married)
b. Married or common-law relationship
c. Separated or divorced
d. Widowed

5. What is your ethnic background?
a. Caucasian
b. Asian
c. African Canadian
d. Hispanic
e. First Nations
f. Other_______________

6. What is your nationality?
a. Canadian
b. American
c. Other_______________

7. Does your partner have a diagnosis of IBD? ☐ Yes ☐ No

8. What is your primary **current** employment status?
a. Student
b. Retired
c. Unemployed
d. Employed (part-time or full-time)
e. Never employed

If you described yourself as "unemployed", "retired", or "never employed", please go to Q10

9. If you are currently employed, what type of job classification best describes your job?
a. Administrative (e.g. teacher, nurse, accountant, admin support etc.)
b. Blue Collar (carpenter, mechanic, general laborer etc.)
c. Other

10. Please specify your current level of annual income (before taxes):
a. Prefer not to say d. $40,000 - $69,900
b. Less than $20,000 e. $70,000 - $99,900
c. $20,000 - $39,900 f. $100,000 or more

11. What is the highest level of education that you have completed?
a. less than high school diploma
b. completed high school diploma
c. completed trade, technical, vocational or business school
d. completed university undergraduate degree

e. completed post-graduate degree

12. Have you ever been diagnosed with depression?

☐ Yes, I was diagnosed with depression **before my IBD diagnosis**

☐ Yes, I was diagnosed with depression **after my IBD diagnosis**

☐ No, I have never been diagnosed with depression

13. Have you ever been diagnosed with anxiety?

☐ Yes, I was diagnosed with anxiety **before my IBD diagnosis**

☐ Yes, I was diagnosed with anxiety **after my IBD diagnosis**

☐ No, I have never been diagnosed with anxiety

14. Have you experienced any of the following in the last 6 months? Please circle all that apply:

a. Stress

b. Fatigue

c. Malaise

d. Anxiety

e. Depression

f. Other: ____________________

15. Do you have a history of any other mental health illness (ie. other than depression or anxiety) that has required medication or other form of therapy?

☐ Yes

☐ No

If yes, please specify the illness ______________________________

16. Do you have any other medical conditions **unrelated** to Ulcerative Colitis or Crohn's Disease or Inflammatory Bowel Disease (IBD) of undetermined type?

☐ Yes

☐ No

If yes, please specify:

i. _________________________

ii. _________________________

iii. _________________________

iv. _________________________

v. _________________________

------------------------------------ END OF SECTION A ------------------------------------

SECTION F – Pain History

1. Which best describes any pain that you have related to IBD?
a. Never have any pain with IBD flares or in between flares.
b. Only have pain with IBD flares and pain-free in between flares
c. Constant pain in between flares and increased pain during IBD flares
d. Constant pain that changes for different reasons and also with IBD flares
e. Constant pain that never changes with or without IBD flares

2. If you do have pain, **over the last 2 weeks** where has it been located (circle all that apply):
a. Head/Neck e. Legs
b. Back f. Joints Only
c. Abdomen/Belly g. Everywhere at different times
d. Arms e. No pain in the last 2 weeks

3. In the **last 2 weeks**, on a scale of 0-10, what was your average pain rating:
0 1 2 3 4 5 6 7 8 9 10
No pain Worst pain

4. Have you **ever** used any type of pain killer for relief of your abdominal/belly pain (for example, codeine, morphine, tylenol/acetominophen, ibuprofen or non-steroidal antiinflammatory
drugs / NSAIDs) **for more than one month**?
☐ Yes
☐ No
If yes, please specify which medications:
Nam e Dose Frequency (# of times/day)
1.
2.
3.
4.
5.

5. Have you been using pain killers **every day for the last 30 days or more**?
☐ Yes
☐ No
If yes, please specify which medications:
a. Same as chart above OR
b. Current pain killer use:
Nam e Dose Frequency (# of times/day)
1.
2.
3.
4.
5.

6. Have you been using pain killers **during your current pregnancy, if applicable?**
☐ Yes
☐ No
If yes, please specify which medications:
a. Same as chart above OR
b. Current pain killer use:
Name Dose Frequency (# of times/day)

1.
2.
3.
4.
5.

------------------------------------- END OF SECTION F -------------------------------------

SECTION G - Substance Use

1. How would you describe your tobacco smoking history?
a. Never smoked regularly
b. Currently smoke regularly
c. Used to smoke but I have quit (year quit:)
d. Used to smoke, but have temporarily quit for pregnancy (year quit:)
2. If you currently smoke, how many packs do you smoke per day: packs/day
3. **Over the last 2 weeks**, how much alcohol did you consume per week (1 beer=1 wine glass= 1 ounce hard liquor)?: drinks/week
4. Have you ever used an illicit or illegal substance such as LSD, heroin, cocaine, etc. (**not** including cannabis) outside of pregnancy?
☐ Yes
☐ No
5. Have you used an illicit or illegal substance such as LSD, heroin, cocaine, etc. (**not** including cannabis) during any pregnancy, if applicable?
☐ Yes
☐ No
☐ N/A

------------------------------------ END OF SECTION G ------------------------------------

SECTION H - CANNABIS USE

Please answer the following questions, regardless of your current pregnancy status

1. Which response best describes your intention to use Cannabis in the future?
a. None
b. Maybe
c. Probably
d. Definitely

2. What is your understanding of Cannabis use during pregnancy? (circle all that apply)
a. Cannabis is safe throughout pregnancy and can be continued
b. Cannabis is safe only in the first trimester
c. Cannabis is safe only in the second trimester
d. Cannabis is safe only in the third trimester
e. Cannabis is not safe during pregnancy.

3. What is your understanding of Cannabis use and its effect on the fetus? (circle all that apply)
a. Cannabis use has no effect on the fetus and is safe
b. Cannabis use can restrict oxygen to the fetus
c. Cannabis use can cause respiratory problems such as asthma
d. Cannabis use can cause immune disorders such as eczema
e. Cannabis use can affect the baby's brain development

4. Have you discussed cannabis use during pregnancy ☐ Yes
with a health-care provider? ☐ No
i. **If yes**, did your health-care provider discuss ☐ Yes
any potential concerns and risks of cannabis ☐ No
use during pregnancy?

5. Would you be interested in participating in ☐ Yes
a clinical trial (a study where the effectiveness ☐ No
is measured) of Cannabis? ☐ Maybe

6. Do any of your immediate family members ☐ Yes
(father, mother, sister, brother) also use Cannabis? ☐ No

7. Have you ever used Cannabis ☐ Yes (**If YES**, please complete the following table)
(marijuana, 'pot', 'hash') ☐ No (**If NO**, Please skip to **SECTION J**, Page 16)
